# Spatial–temporal variations of river water quality under human-induced land use changes in large river basins

**DOI:** 10.1038/s41598-025-20876-z

**Published:** 2025-10-22

**Authors:** Xiaojing Zhang, Bing Yu, Zhuohang Xin, Ming Cong, Chi Zhang

**Affiliations:** School of Infrastructure Engineering, Dalian University of Technology, Dalian, 116024 China

**Keywords:** Land use patterns, Seasonal variation, Water quality, Human health, Environmental impact, Risk factors

## Abstract

Land use patterns and anthropogenic activities exert diverse impacts on river water quality and ecosystem health across spatial and temporal scales. Under ongoing pressures from human activities and climate change, it is urgently needed to understand the pollution mechanisms and associated ecological risks in surface waters through cross-basin assessments that incorporate a broad set of water quality parameters. This study investigated the spatial–temporal variations of 22 water quality parameters, including physico-biochemical indicators, nutrients, and heavy metals, at 39 sampling sites across the Daliao River (DLR), Shuangtaizi River (STR), and Naoli River (NLR). Field observations were conducted in September 2019, December 2020, and June 2020, covering the wet, dry, and agricultural seasons to assess the influencing mechanisms of natural factors and human activities on river water quality and human health. Principal Component Analysis revealed a notable seasonal variation in water quality components for DLR and STR, which indicated substantially high concentrations of TN, NO_3_^-^ and NH_4_^+^ in the dry season. The heavy metal risk for children was calculated at 8.44E-05 year^-1^ in NLR during the agricultural season, which exceeded the maximum acceptable limit, with carcinogenic arsenic being the primary contributor. Redundancy analysis (RDA) was employed to examine the influence of land use patterns on water quality across different seasons and spatial scales. DO and COD showed a strong correlation with dry land and woodland, while nutrients and Chl-a were strongly correlated with paddy fields and building areas. This study underscores the critical role of land use management in harmonizing development with ecological sustainability and provides scientific evidence supporting the achievement of Sustainable Development Goal 6 (SDG 6), which focuses on clean and safe water.

**Supplementary Information:**

The online version contains supplementary material available at 10.1038/s41598-025-20876-z.

## Introduction

Water quality in river systems is a global concern that affects aquatic ecosystems, human health, and socio-economic development^1,2^. Both natural and anthropogenic factors influence river water quality, with urbanization and agricultural runoff identified as primary drivers of its degradation^3,4^. The rapid expansion of these activities has altered land use patterns, leading to the introduction of diverse pollutants that degrade river water quality through multiple pathways and mechanisms^5^. River water pollution poses serious ecological and public health risks. Excessive nutrient loading leads to eutrophication, triggering algal blooms and oxygen depletion, while even low concentrations of heavy metals can bioaccumulate in aquatic organisms, potentially causing severe health effects such as neurological impairment and carcinogenesis^6,7^. These challenges underscore the urgent need for integrated land use and water quality management strategies to safeguard ecosystem integrity and ensure clean water access for human populations.

The response of surface water environments to natural processes and anthropogenic disturbances has been extensively investigated over the past decades^8^. Natural factors such as water temperature, precipitation, topography, and soil properties play a vital role in shaping river water quality. Seasonal variations in rainfall regulate surface runoff processes, which in turn determine the mobilization and transport of pollutants into aquatic systems^9^. Moreover, watershed features such as vegetation cover and slope affect the retention and dispersion of pollutants, intensifying spatial heterogeneity in water quality patterns^10^. Natural factors regulate the transport and retention of pollutants within river systems, whereas anthropogenic activities substantially increase external pollutant loads^11^. Agricultural practices, particularly the application of fertilizers and pesticides, substantially contribute to nutrient loading in rivers^12^. Likewise, industrial effluents may introduce heavy metals and hazardous chemicals into aquatic systems, posing long-term environmental and health risks^13^. Urbanization intensifies these challenges by escalating the wastewater influx into rivers, which often carries elevated levels of organic matter and pathogens^14^. While previous studies have made substantial progress in understanding the factors influencing river water quality, a more comprehensive investigation is needed to address the complex, multi-factorial responses of river water systems to ongoing climate change and intensifying anthropogenic activities.

As human activities continue to modify land use structures within watersheds, the resulting landscape changes increasingly dictate the modes and magnitudes of pollutant delivery into aquatic environments. Land use and landscape patterns are increasingly recognized as key drivers of river water quality, attracting considerable academic attention within the field of water environment research^15^. The expansion of urban land and the intensification of agricultural land use significantly increase the flux of pollutants, such as nitrogen and phosphorus, into rivers, while forests and wetlands improve water quality through processes such as retention, adsorption, and denitrification^16^. Studies have found that the response of river water quality to land use is generally non-linear, with water quality significantly deteriorating when the proportion of arid farmland exceeds 54%^17–19^. Furthermore, land use influences water quality through the distinct modes of pollutant entry into rivers associated with human activities. Pollution in urban areas is largely attributable to point sources, whereas agricultural pollution mainly originates from non-point sources, which are often inadequately controlled^20^. As a result, the impact of land use types on water quality demonstrates significant variability across different temporal and spatial scales. Integrating data from multiple basins would advance understanding of how land use differentially influences water quality parameters.

Land use patterns significantly influence river water quality by contributing heavy metals through untreated mining residues, industrial wastewater, and agricultural runoff, ultimately posing risks to human health^21^. Notably, arsenic, lead, and mercury exhibit high toxicity even at very low concentrations^22^. As non-degradable substances, they can accumulate in the human body, causing damage to the nervous system and internal organs^23^. Understanding the dynamics of heavy metals in surface water is crucial, especially given their sensitivity to land use patterns that can significantly influence their distribution and potential risks to human health.

Building on previous research, this study investigated the spatial–temporal variability of multiple pollutants and their associated health risks to elucidate how seasonal anthropogenic practices and land use patterns influence pollutant dynamics. The Songliao River Basin, characterized by diverse land use and urban development patterns, consequently exhibits notable differences in water contamination levels. However, conventional water quality management typically relies on single-indicator methods, which inadequately characterize the complex interactions among multiple pollutants under varying land use changes and human activities. This study conducted a comprehensive assessment of water quality in three representative rivers of the Songliao River Basin: the Daliao, Shuangtaizi, and Naoli Rivers, which reflect varying degrees of anthropogenic influence and distinct land use patterns. The main objectives are: (1) to compare the spatial and temporal variations in physico-biochemical parameters, nutrients, and heavy metals across three rivers in the Songliao River Basin; (2) to provide a concise evaluation of water quality status and human health risk with heavy metals; (3) to assess multi-scale land use impacts on water quality and identify the primary factors driving regional variation. Under the sustained pressure of intense anthropogenic activities, this study provides valuable insights to inform targeted and differentiated river management strategies.

## Materials and methods

### Study area

**Fig. 1:**
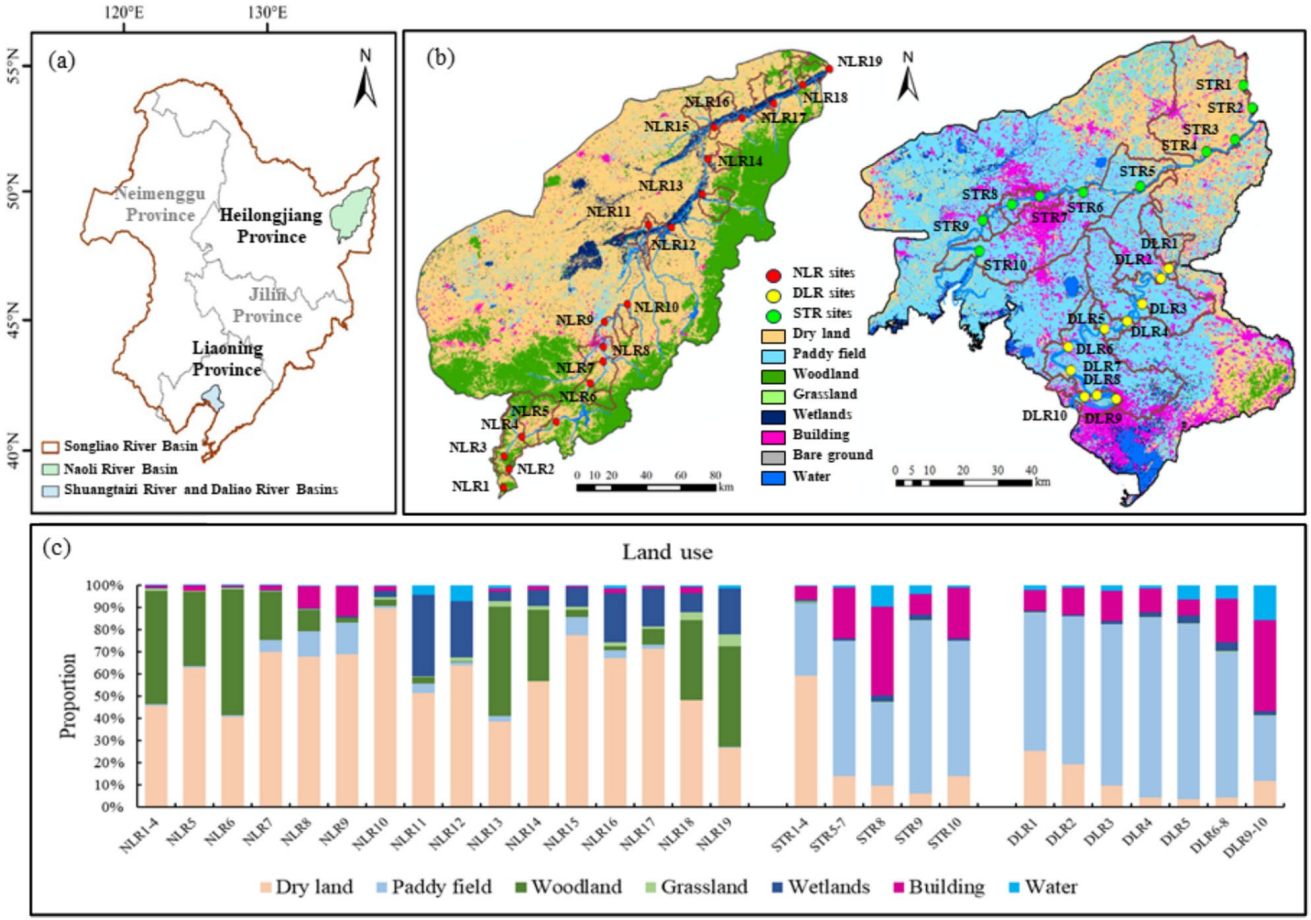
Map showing (**a**) the locations of the Naoli River Basin, Shuangtaizi River Basin and Daliao River Basin, (**b**) locations of sampling sites and land use patterns of the basins. (**c**) the proportions of land uses within the drainage area of each sampling site.

The Songhua River-Liao River Basin (hereafter Songliao River Basin) in northeast China is a vital industrial and agricultural base for the country^24^. This study focuses on three regions within the basin, namely the Daliao River (DLR) and Shuangtaizi River (STR) of the Liao River (LR) Basin in the south, and the Naoli River (NLR) of the Songhua River Basin in the north. The DLR and STR basins jointly cover an area of approximately 945km^2^ (Fig. 1b), and serve as key centers of economic activity and human settlement in Northeast China. The Naoli River (NLR) spans 596 km and flows through a vital grain-producing region of China (Fig. 1a). The DLR, STR and NLR all experience a northeast monsoon climate, with long, cold winters and warm, rainy summers (Fig S1). Mean annual air temperatures range from −10 °C to 30 °C in the STR and DLR, and from −20 °C to 25 °C in the NLR, with July and January being the hottest and coldest months, respectively. Annual precipitation is 600–1,000 mm in the STR and DLR, and 500–800 mm in the NLR. For all basins, precipitation is concentrated in August, while the dry season typically spans from December through February.

The DLR, STR, and NLR basins show distinct spatial heterogeneity in terms of urbanization level, water use intensity and land use patterns. Despite their geographical proximity, the DLR and STR basins differ markedly in urbanization and population density, with approximately 3,080 and 550 people/km^2^, respectively. The NLR Basin is a vast and sparsely populated region, with a low population density of 26 people/km^2^. According to the 2020 Songliao River Basin Water Resources Bulletin, the average annual agricultural water use in the NLR Basin was approximately 57,700 m^3^/km^2^, while domestic water use was 2,700 m^3^/km^2^. In contrast, the LR Basin reported an agricultural water use of 47,700 m^3^/km^2^ and domestic use of 12,400 m^3^/km^2^.

Land use data were derived from the 2020 dataset published by the Aerospace Information Research Institute, Chinese Academy of Sciences, with a spatial resolution of 30 m (Fig. 1b). The NLR Basin is dominated by dryland, woodland, and wetlands, accounting for 63.0%, 25.0%, and 8.3% of the total basin area respectively, while paddy field and building areas cover only 3.1% and 2.4%. The STR Basin is primarily composed of paddy fields (49.0%), dry land (30.8%), and building areas (16.4%), and the DLR Basin is primarily composed of paddy fields (61.7%) and building areas (19.3%). To assess the impact of human activities at each sampling site, the corresponding drainage areas were delineated using ArcGIS hydrological tools, and the land use composition within each was subsequently quantified (Fig. 1c). In the NLR Basin, woodland within drainage areas of sampling sites generally declined from upstream to midstream, accompanied by increasing dryland and urban land areas. Downstream areas exhibited increased woodland and wetland coverage with corresponding dryland contraction. In the STR Basin, land use shifts from dryland-dominated agriculture (59.3%) in the upper reaches to intensive urban land use (40.1%) at midstream sites, followed by rice-dominated paddy fields (60.0%) at downstream sites. In the DLR Basin, most sampling sites are dominated by rice cultivation, with paddy fields covering 69.7% of delineated drainage areas, whereas the downstream sites (DLR9 and DLR10) are primarily characterized by urban land use, accounting for 40.9%. Given the distinct land use patterns, agricultural practices and levels of human activities among these three rivers, the spatial and temporal variations in both the pollution types and levels of river pollutants are likely to occur.

### Water sampling and testing

We sampled a total of 39 sites, with 10 in the Daliao River (DLR1-DLR10), 10 in the Shuangtaizi River (STR1-STR10), and 19 in the Naoli River (NLR1-NLR19) (Fig. 1). Sampling points were spatially balanced along the upstream, middle, and downstream reaches to capture spatial variability in land use. For example, upper reaches include DLR1-3 (STR1-4, NLR1-7), middle reaches comprise DLR4-6 (STR5-7, NLR8-13), while lower reaches contain DLR7-10 (STR8-10, NLR14-19). Water samples were collected from the surface layer (0–20 cm) at the midstream of each site during three representative periods: the wet season (September 2019), dry season (December 2020), and the agricultural season (June 2020). A total of 117 surface water samples (0–20 cm depth) were collected using pre-cleaned high-density polyethylene bottles. Each 1 L water sample was immediately filtered through a 0.45 μm membrane filter and stored at 4 °C for subsequent analysis of physico-biochemical parameters, nutrients, and heavy metals. All measurements were performed in triplicate to ensure accuracy and reproducibility.

Water temperature (WT), pH and dissolved oxygen (DO) concentration were measured on site using an EXO probe (Yellow Spring Instruments, Ohio, United States). Dissolved nutrients (NH_4_^+^, NO_3_^-^, NO_2_^-^, PO_4_^3-^), total nitrogen (TN) and total phosphorus (TP) were analyzed by Continuous Flow Analyzer (CFA, SEAL Auto Analyzer 3). Heavy metals were analyzed by Inductively Coupled Plasma—Optical Emission Spectrometry (ICP-OES, Thermo) and the detection limit is 0.01 ppm. Permanganate index (COD_(Mn)_) was measured using the potassium permanganate method. Total Suspended Solids (TSS) was determined by the weight difference of a Whatman GF/F filter (pore size 0.7 μm) after 2 h of drying at 105℃. Chlorophyll-a was determined using the 90% acetone extraction method, followed by spectrophotometry at absorbances at 664, 647, 630, and 750 nm.

### Data analysis and assessment

#### Statistical analysis

Pearson’s correlation coefficient was used to assess correlations between water quality parameters. Principal Component Analysis (PCA) was used to identify the principal water quality components and the main parameters influencing them. Redundancy Analysis (RDA) was conducted in Canoco 5.0 to investigate the seasonal relationships between land use types and water quality parameters across the three studied rivers. This method enables the simultaneous analysis of multiple response and explanatory variables, with the vector lengths and angles in the ordination plot reflecting the strength and direction of their interactions^25^.

#### Canadian Council of Ministers of the Environment Water Quality Index (CCME-WQI)

The CCME-WQI is an objective-based index that is typically calculated using three components: F1, F2, and F3^26^. It is widely used in water quality evaluation^27^, and has been shown to be suitable for waters in China^28^. The index values are calculated as follows:

1 $$CCME WQI=100-\frac{\sqrt{{F}_{1}^{2}+{F}_{2}^{2}+{F}_{3}^{2}}}{1.732}$$

2 $${F}_{1}=\frac{P}{N}\times 100$$

3 $${F}_{2}=\frac{q}{M}\times 100$$

4 $${F}_{3}=\frac{Q}{0.01Q+0.01}$$

5 $$Q=\frac{\sum S}{M}$$

where F_1_ represents the percentage of the selected parameters that exceed the objectives; P is the number of water quality parameters exceeding the objectives; N is the total number of selected parameters. F_2_ represents the percentage of the number of times individual tests exceed the objectives during the considered time period; q is the number of failed tests; M is the total number of tests. F_3_ represents the amplitude of deviation by which failed test values do not meet the objectives; S is the exceeding range of a failed test; and Q is the normalized parameter.

The CCME-WQI value ranges from 0 to 100 and is classified into five categories: Excellent (95–100), Good (80–94), Fair (65–79), Marginal (45–64), and Poor (0–44). In this study, water quality parameters (criteria) for CCME-WQI evaluation included DO (5 mg/L), COD_(Mn)_ (6 mg/L), NH_4_^+^ (1 mg/L), TN (1 mg/L), TP (0.2 mg/L), Chl-a (10 μg/L), Zn (1 mg/L), Pb (0.05 mg/L), Mn (0.1 mg/L), Ni (0.02 mg/L). All criteria were based on the China’s Environmental Quality Standard for Surface Water, Class III^29^ and the Standards for Drinking Water Quality^30^. These 10 parameters were selected based on principal component analysis and correlation analysis to capture the main pollution patterns of the study area and to minimize potential multicollinearity among parameters.

#### Human health risk assessment

Human health risk assessment is used to evaluate the risks associated with heavy metal exposure. Metals can enter the human body through oral intake, inhalation, and dermal absorption^31^, with the latter two occurring at levels 2–3 orders of magnitude lower than oral intake and thus can be disregarded. The United States Environment Protection Agency^32^ water environment health risk assessment model was employed to assess the hazards of heavy metals in the surface water through oral intake, with separate calculations for adults and children. The models included evaluations for both carcinogenic and non-carcinogenic risks.

The carcinogen evaluation model (R^c^) is calculated as follows^17,18,33^:

6 $${R}^{c}={\sum }_{i=1}^{k}{R}_{ig}^{c}={\sum }_{i=1}^{k}\frac{1-{exp}^{(-{D}_{ig}{Q}_{ig})}}{W}$$

7 $${D}_{ig}=\frac{A\times {C}_{i}}{B}$$

The non-carcinogen evaluation model (R^n^) is calculated as follows^17,18,34^:

8 $${R}^{n}={\sum }_{j=1}^{m}{R}_{jg}^{n}={\sum }_{i=1}^{m}\frac{{D}_{jg}\times {10}^{-6}}{{RfD}_{jg}\times W}$$

9 $${D}_{jg}=\frac{A\times {C}_{j}}{B}$$

where *D*_i/jg_ is the average intake daily dose through ingesting carcinogens [mg/kg/day]; *C*_*i/j*_ is the concentration of a particular pollutant (mg/L); A represents the ingestion rate (L/day; A = 2.2L/day for adults and 1.0L/day for children); *B* represents the average body weight (kg; *B* = 56 kg for adults and 22 kg for children)^35,36^ *W* represents average exposure time of locals (years; *W* = 75.98 years in NLR Basin; *W* = 76.38 in LR Basin); *Q*_*ig*_ is the cancer slop factor (mg/kg/day) and *RfD*_*jg*_ is reference dosage for noncarcinogenic pollution (mg/kg/day), respectively, were obtained from the database of the integrated risk information system^37^.

The total health risk evaluation is calculated as follows:

10 $${R}_{total}={R}^{c}+{R}^{n}$$

where ${R}^{c}$ represents the health risk value of chemical carcinogens(year^−1^); ${R}^{n}$ represents the health risk value of non-chemical carcinogens(year^−1^); ${R}_{total}$ represents the total health risk value (year^−1^).

## Results

### Physico-biochemical parameters and nutrients

Fig S2 displays the measured physico-biochemical parameters in DLR, STR and NLR during the wet, dry, and agricultural seasons. For DO, NLR had higher values during the dry season and lower values during the agricultural season, with mean values of 13.54 mg/L and 5.99 mg/L, respectively. In contrast, in DLR and STR, DO values were higher during the agricultural season with mean values of 6.08 mg/L and 8.42 mg/L, respectively, and lower during the dry season, with mean values of 5.80 mg/L and 4.99 mg/L, respectively. The temporal variation of COD_(Mn)_ in NLR fluctuated obviously, with mean values of 9.45 mg/L, 18.8 mg/L and 0.82 mg/L in the wet, dry and agricultural seasons, respectively. The COD_(Mn)_ value in DLR was lower in wet season (mean value 1.76 mg/L) and higher during agricultural season (mean value 5.2 mg/L), and that in STR was stable (mean value 5.56 mg/L). In the DLR and STR, Chl-a concentrations peaked in the agricultural season, ranging from 4.53 to 14.27 μg/L and 8.38 to 25.59 μg/L, respectively, reflecting heightened algal growth. Significant spatial variation was observed along the river reaches, with higher Chl-a values recorded at midstream sampling sites DLR5 and STR5 in both rivers. Chl-a concentrations were slightly higher in the wet season compared to the dry season, yet consistently remained below 10 μg/L, indicating no evidence of eutrophication. In contrast, Chl-a concentrations in the NLR remained low across all seasons, ranging from 0.39 to 3.90 μg/L.

**Fig. 2:**
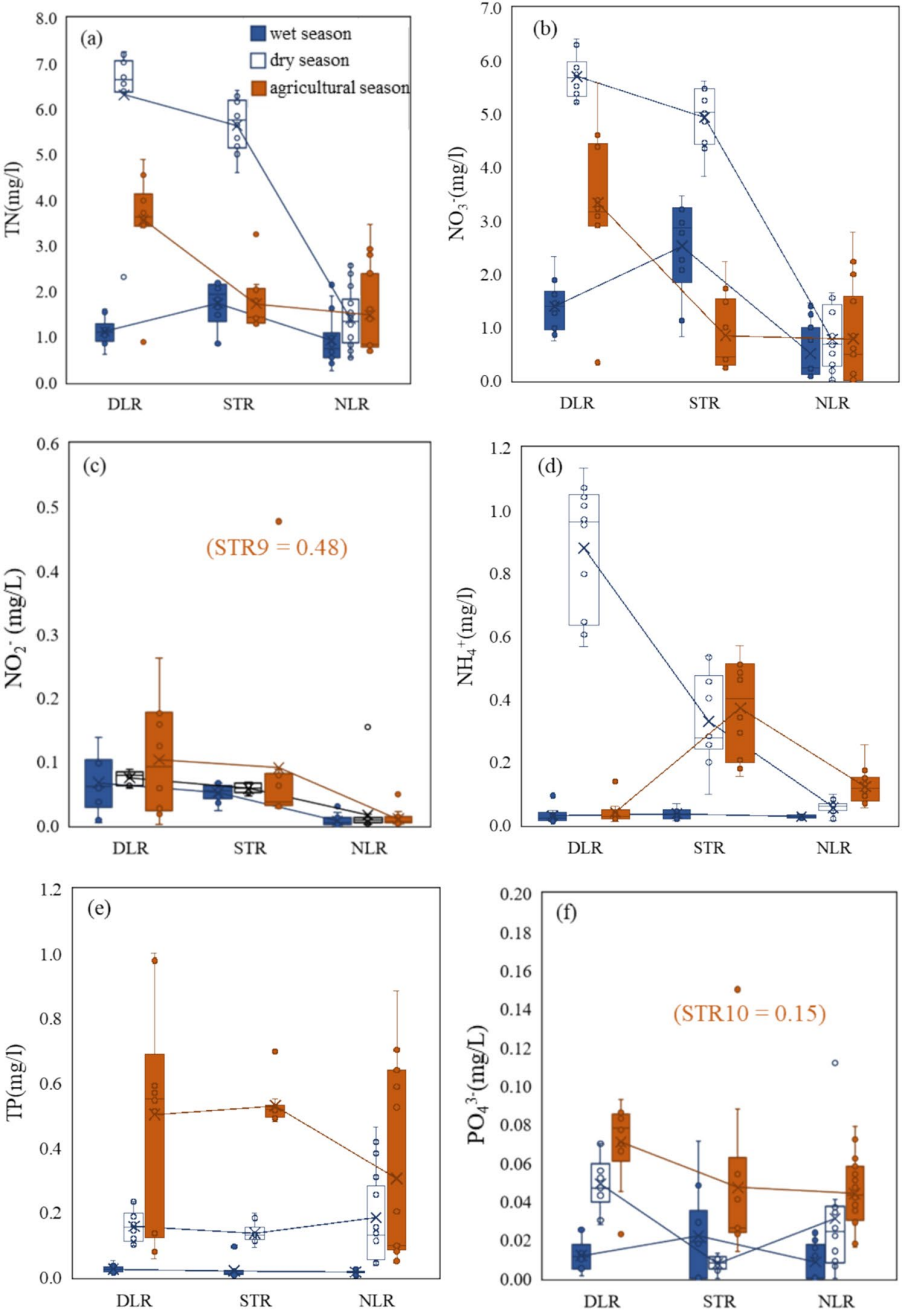
Box charts showing the nutrient concentrations during wet, dry and agricultural seasons in DLR, STR and NLR.

The nitrogen and phosphorus concentrations across different periods are shown in Fig. 2. DLR and STR exhibited obviously higher TN concentrations during the dry season, with average values reaching 6.3 mg/L and 5.6 mg/L, respectively. In contrast, NLR had its higher TN concentrations during the agricultural season, peaking at 3.47 mg/L. All three rivers showed their lowest TN concentrations during the wet season. In terms of inorganic nitrogen compounds, NO_3_^-^ (Fig. 2b) showed a similar trend to TN across different periods. NLR consistently exhibited little variation in NO_3_^-^ levels across the three periods, with concentrations of 0.5 mg/L in wet, and 0.8 mg/L in both the dry and agricultural seasons. The concentrations of NO_2_^-^ and NH_4_^+^ were generally low (Fig. 2c, d). NO_2_^-^ values were relatively high during the agricultural season in all three rivers. Except for specific points such as STR9 reaching 0.48 mg/L and DLR2 reaching 0.26 mg/L, the values at other points were all below 0.2 mg/L. This notably high value at STR9 was likely due to intensified nitrification and incomplete denitrification under conditions of high nitrogen input and limited oxygen availability. In DLR, NH_4_^+^ concentrations were higher during the dry season, reaching 0.9 mg/L, while remaining relatively low during other periods. Overall, nitrogen concentrations in the LR Basin were higher than in the NLR Basin, with the relatively high nitrogen levels occurring during the dry season.

Notably, TP and PO_4_^3-^ concentrations in all three rivers were obviously elevated during the agricultural season (Fig. 2e, f), indicating the pronounced impact of fertilization on phosphorus pollution. Additionally, TP concentrations in DLR and NLR during the agricultural season exhibited more dispersed, ranging from 0.06 to 1 mg/L and 0.04 to 0.88 mg/L, respectively, suggesting a strong spatial heterogeneity in TP distribution. TP concentrations were obviously higher in the dry season than in the wet season, with mean values of 0.16 mg/L, 0.14 mg/L, and 0.18 mg/L in the dry season, and 0.02 mg/L, 0.02 mg/L, and 0.01 mg/L in the wet season for DLR, STR, and NLR, respectively. PO_4_^3-^ concentrations were relatively low in the three rivers, with the highest levels also occurring during the agricultural season. The highest value (0.15 mg/L) occurred at the downstream site STR10, likely due to cumulative PO_4_^3-^ accumulation along the river.

### Heavy metals

The superposition values of heavy metal concentration at all sampling sites are shown in Fig S3. In NLR, the values were notably higher at NLR6 (0.17 mg/L), NLR10 (0.26 mg/L) and NLR17 (0.26 mg/L), primarily due to contributions from Mn and Mo. The total concentrations of the remaining NLR sites were relatively low, ranging from 0.03 to 0.07 mg/L, with As, Zn, Mo and Ni being the main contributors. In DLR, a notable high value was detected at DLR10 (0.24 mg/L) near the estuary, while it ranged from 0.08 to 0.11 mg/L at the remaining sites of DLR1 to DLR9. The total concentrations in STR ranged from 0.06 to 0.13 mg/L. Mo, Mn, Zn, Pb and Ni were five contributors to total concentrations in DLR and STR. NLR exhibited relatively low metal content compared to DLR and STR, with exceptions of considerable high values at three sites.

Table 1 shows the statistics of heavy metal concentrations in three rivers. The standard values were based on the Environmental Quality Standards for Surface Water (MEE, 2002). In all rivers, Cr, As, Cd, Cu, and Zn met their respective standards, with some values marked as " < 0.01 mg/L" indicating concentrations below the ICP-OES detection limit (0.01 mg/L). These values indicated that the heavy metal concentrations did not exceed regulatory standards, and no further statistical adjustments were applied. In DLR, Pb, Mn and Mo exceeded the standard limits, with excessive rates of 3.3%, 10% and 6.7%, respectively. In STR, Mo and Ni were above the standards, both with an exceedance rate of 3.3%. In NLR, Mn, Mo, and Se exceeded the standard thresholds, with exceedance rates of 8.1%, 2.7%, and 2.7%, respectively.

**Table 1:** Statistics of heavy metal concentrations and excessive rates in DLR, STR and NLR.

Item		Cr	As	Cd	Pb	Cu	Zn	Mn	Mo	Se	Ni
Standard limit (mg/L)		0.05	0.05	0.005	0.05	1.00	1.00	0.10	0.07	0.01	0.02
DLR	Mean value (mg/L)	< 0.01	< 0.01	< 0.01	0.02	< 0.01	0.03	0.02	0.04	< 0.01	0.01
	Minimum value (mg/L)	< 0.01	< 0.01	< 0.01	< 0.01	< 0.01	< 0.01	< 0.01	0.01	< 0.01	< 0.01
	Maximum value (mg/L)	< 0.01	< 0.01	< 0.01	0.15	< 0.01	0.22	0.12	0.21	< 0.01	0.01
	Excessive rate (%)	0	0	0	3.3	0	0	10	6.7	0	0
STR	Mean value (mg/L)	< 0.01	< 0.01	< 0.01	0.01	< 0.01	0.03	0.01	0.03	< 0.01	0.01
	Minimum value (mg/L)	< 0.01	< 0.01	< 0.01	< 0.01	< 0.01	< 0.01	< 0.01	0.01	< 0.01	< 0.01
	Maximum value (mg/L)	< 0.01	0.01	< 0.01	0.03	< 0.01	0.08	0.03	0.16	0.01	0.04
	Excessive rate (%)	0	0	0	0	0	0	0	3.3	0	3.3
NLR	Mean value (mg/L)	< 0.01	< 0.01	< 0.01	< 0.01	< 0.01	0.02	0.02	0.03	< 0.01	0.01
	Minimum value (mg/L)	< 0.01	< 0.01	< 0.01	< 0.01	< 0.01	< 0.01	< 0.01	< 0.01	< 0.01	< 0.01
	Maximum value (mg/L)	< 0.01	0.05	< 0.01	0.02	0.01	0.06	0.24	0.47	0.03	0.02
	Excessive rate (%)	0	0	0	0	0	0	8.1	2.7	2.7	0

### Correlation and principal component analyses

Table S1 shows the correlation matrix among 20 water quality parameters across all sampled sites and periods. TN and NO_3_^-^ exhibited a strong correlation of 0.942 (P < 0.01), indicating that they were closely correlated pairs, which suggested a very high similarity in their sources. WT showed significant correlations with pH, DO and other 12 parameters (P < 0.01).

**Fig. 3:**
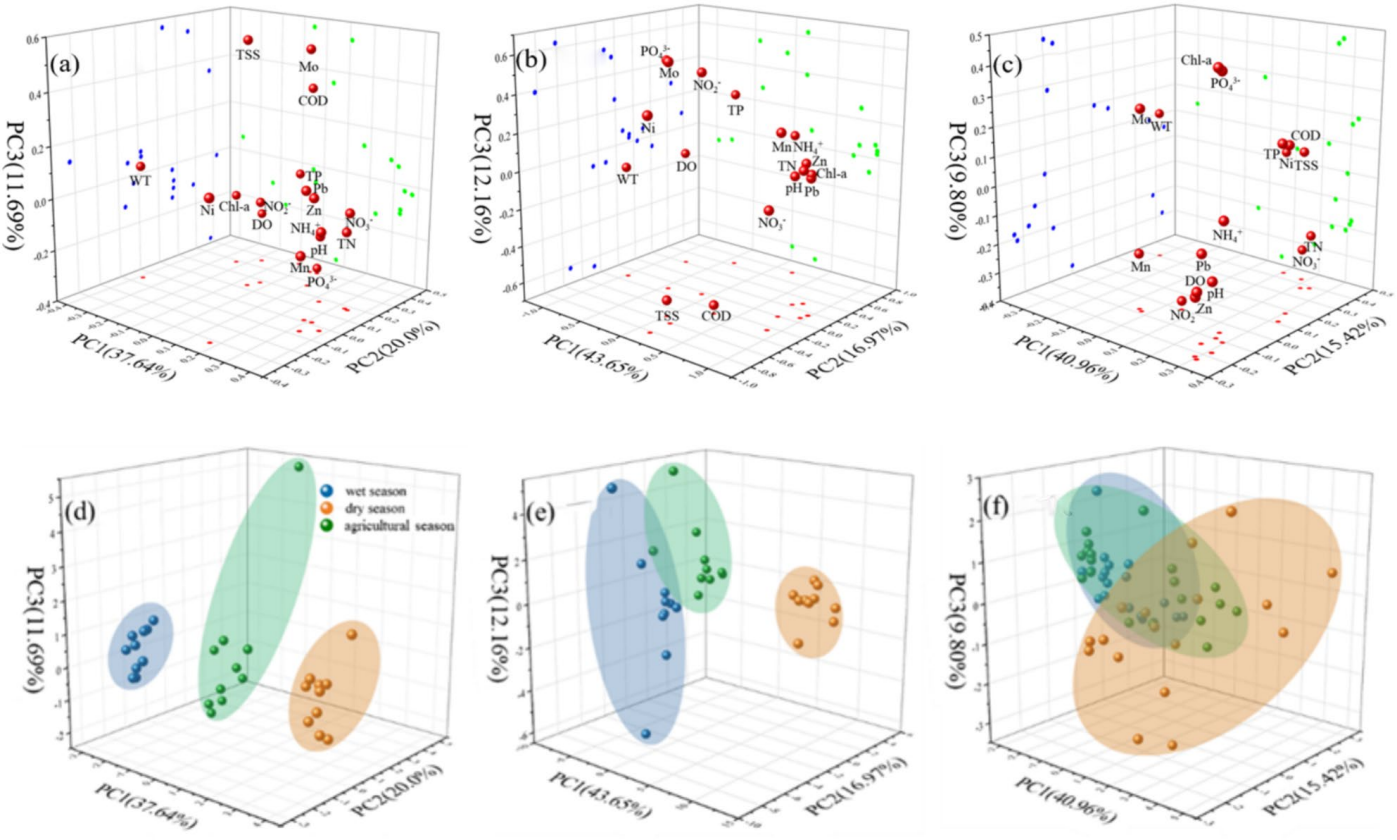
PCA’s loading plots of (**a**) DLR, (**b**) STR, and (**c**) NLR and the score plots of (**d**) DLR, (**e**) STR, and (**f**) NLR.

PCA was performed to identify key parameters influencing water quality in the three rivers, using the Kaiser–Meyer–Olkin (KMO) test for sampling adequacy and Bartlett’s test for significance, along with scree plots to determine the number of principal components (Table S2, Fig S4). Figure 3 shows the principal component loading plots and score plots for the three rivers. Three principal components (PCs) were retained, with their cumulative variance exceeding 65% of the total variance. In DLR (Fig. 6a), PC1 accounted for 37.64% of the total variance, which had a negative loading on WT (−0.39), and positive loadings on NH_4_^+^ (0.38), NO_3_^-^ (0.35), TN (0.33), Mn (0.33), and Zn (0.32). These variables were the main contributors to PC1, indicating that DLR was primarily influenced by nitrogen compounds and heavy metals of Zn and Mn. Nitrogenous compounds in water are often linked to agricultural activities, particularly the use of fertilizers, which contribute significantly to nutrient loading in aquatic systems^38^. The presence of heavy metals like Zn and Mn further indicated potential industrial sources or mining activities contributing to the contamination^39^. PC2 accounted for 20.0% of the total variance, mainly composed of TP (0.42), COD (0.38), DO (0.35) and Ni (0.32). This suggested that PC2 was likely indicative of eutrophication, because elevated levels of TP drove algal blooms, subsequently increasing COD and altering DO levels^40^. PC3 accounted for 11.69% of the total variance, primarily composed of TSS (0.56) and Mo (0.53), which were likely associated with soil erosion and precipitation^41,42^. In STR (Fig. 6b), PC1, PC2 and PC3 explained 43.65%, 16.97% and 12.16% of the total variance, respectively. Similar to DLR, PC1 showed a negative loading on WT (−0.36) and positive loadings on Pb (0.36), Zn (0.36), TN (0.35) and NO_3_^-^ (0.33). PC2 was mainly influenced by high loadings of NH_4_^+^ (0.45) and TP (0.38), while other heavy metals, biological and chemical parameters contributed to PC3. In NLR (Fig. 6c), PC1 and PC2 accounted for 40.96% and 15.42% of the total variance, respectively, while PC3 explained 9.76%. PC1 comprised TP (0.32), pH (0.31), DO (0.31), COD (0.31), WT (−0.30), NH_4_^+^ (0.29), Zn (0.29) and Pb (0.25).

Figures 3d, 3e, and 3f plot the PCA scores of sampling points in wet, dry and agricultural seasons. From Fig. 3d, it was evident that DLR had a distinct clustering pattern along PC1 among the three periods. The PC1 score was the highest in the dry season, with NH_4_^+^, NO_3_^-^, TN, Mn and Pb being identified as the most influential parameters. This reflected that nitrogen and heavy metals were more easily accumulated during this period. In STR, several sampling points from the wet season overlapped with those from the agricultural season, but overall, the three seasons formed distinct clusters along PC1 (Fig. 3e). In NLR, the primary components for the wet season and agricultural season showed overlap (Fig. 3f), indicating that agricultural activities may be the dominant factor influencing these components. In contrast, the dry season was clearly separated from the other two periods, with the primary components more dispersed, suggesting that pollutant sources varied across sampling sites.

### CCME-WQI results

**Fig. 4:**
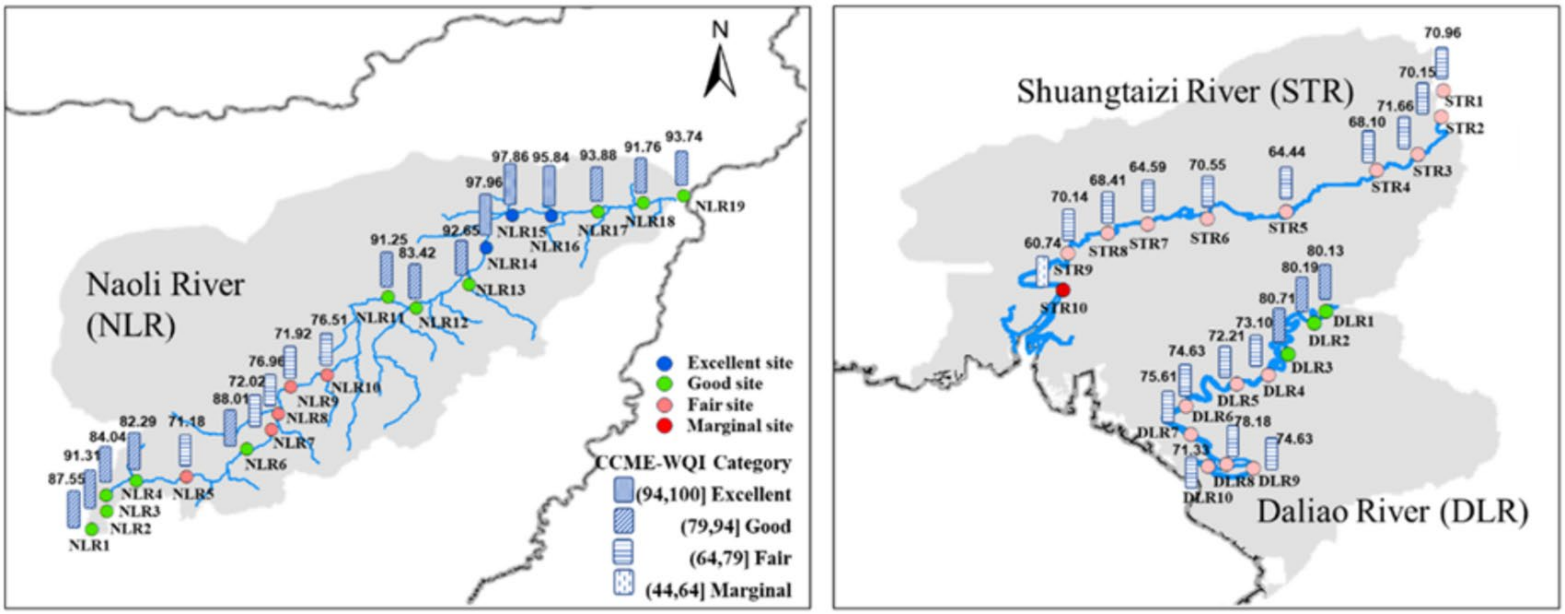
Maps of CCME-WQI evaluation results for sampled sites in NLR, DLR, and STR.

Figure 4 illustrates the water quality categories of CCME-WQI for all sites in DLR, STR, and NLR. Among the 22 parameters, ten parameters (DO, COD_(Mn)_, NH_4_^+^, TN, TP, Chl-a, Zn, Pb, Mn, Ni) were selected for calculating CCME-WQI due to their low inter-correlation and main contribution to the principal components PC1 and PC2, as identified in section "Correlation and principal component analyses". Detailed information on scores of F1, F2, and F3 (Eqs. 2 - 4) and calculated CCME-WQI results are provided in Table S3. Water quality conditions exhibited spatial heterogeneity along the NLR, with three categories identified as excellent, good, and fair (see section "Canadian Council of Ministers of the Environment Water Quality Index (CCME-WQI)" for categories). Sites NLR5 and NLR7-10, located in the upper and middle reaches, were classified as fair, with the lowest WQI value observed at NLR5 (71.18). The remaining upstream and midstream sites (NLR1-4 and NLR11-13) were categorized as good. Downstream sites showed better water quality overall, with excellent conditions at NLR14-16 and good at NLR17-19. The highest value was observed at NLR14 (97.96). In DLR, the CCME-WQI values showed a decreasing trend from upstream to downstream, with the category declining from good to fair. STR exhibited poorer water quality than other rivers. STR1 to STR9 were classified as fair, having values ranging from 64.44 to 71.66. The lowest value was 60.74 at STR10, which belonged to the marginal category. Overall, 74% of sites (14 in number) in NLR were evaluated as good or above, mainly distributed in the upstream and downstream areas, while 26% (5) fell into fair category. In contrast, 85% of sites (17) in DLR and STR were rated as fair or below, with only 15% of sites (3) in the upstream DLR classified as good category.

### Human health risks posed by heavy metals

Table 2 presents the total human health risk values of the three rivers (section "Human health risk assessment"). In comparison, DLR had relatively low health risk values for all three periods, indicating that residents in this area were less affected by heavy metal exposure risks compared to the other two areas. STR exhibited low risk values during the wet season; however, these values increased by four orders of magnitude during the agricultural and dry seasons. In NLR, the health risk values were highest during the agricultural season, followed by the wet season, and declined significantly in the dry season. Due to the lower immune resistance in children, they consistently faced higher heavy metal exposure risks compared to adults.

**Table 2:** Total human health risk values of heavy metals in different periods for the three rivers (year^−1^).

Period	Human	DLR	STR	NLR
Wet season	Adult	2.29E-09	4.05E-09	4.07E-06
	Child	2.65E-09	4.68E-09	4.71E-06
Agricultural season	Adult	4.50E-09	4.45E-05	3.56E-05
	Child	5.21E-09	4.45E-05	8.44E-05
Dry season	Adult	2.17E-08	1.55E-05	4.78E-09
	Child	2.51E-08	1.80E-05	5.53E-09

**Fig. 5:**
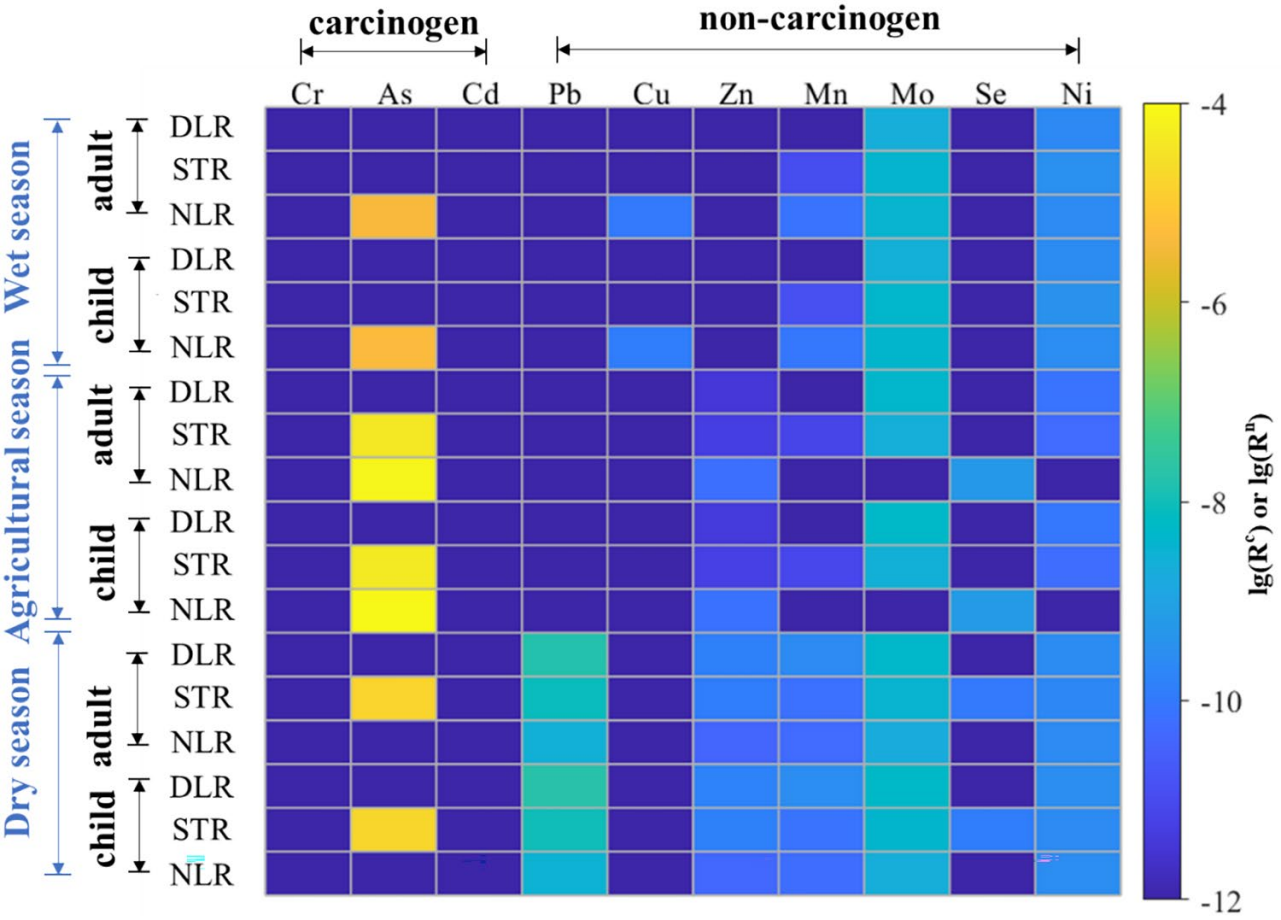
Matrix chart showing adult and child health risks from carcinogenic and non-carcinogenic heavy metals in DLR, STR and NLR during the wet, dry, and agricultural seasons. The matrix values represent the logarithm of the risk values. The color gradient from blue to yellow denotes the increase in risk levels.

Figure 5 illustrates the risk levels of specific metals in the three rivers across different periods, while Table S5 provides detailed risk values. Among all metals, the carcinogenic As posed an evidently high exposure risk to both adults and children, particularly during the wet and agricultural seasons in NLR and the agricultural and dry seasons in STR. Notably, As risk values during the wet season in NLR (Table S5) reached 7.30 × 10^–5^ year^−1^ and 8.44 × 10^–5^ year^−1^, respectively, for adults and children, which exceeded the maximum acceptable limit set by the International Commission on Radiological Protection (ICRP) (5 × 10^–5^ year^−1^). Among non-carcinogenic metals, Mo exhibited relatively higher risk values compared to other non-carcinogens, ranging from 1.78 × 10^–9^ to 5.50 × 10^–9^ year^−1^. The risk values of non-carcinogenic metals were far below the limit value and did not pose a threat to human health. Therefore, the content of the carcinogen As in the study area requires particular attention.

## Discussion

### Spatiotemporal variations of water pollutants in the Songliao river basin

In terms of spatial distribution, the water quality in the NLR Basin was superior to that of the LR Basin. Apart from several sites in the upper and middle reaches classified as fair, water quality across the NLR Basin was predominantly rated as good or excellent. The relatively lower water quality levels in the midstream region may be attributed to the dense urban settlements in this area. Conversely, natural factors including forests and wetlands were likely responsible for enhanced pollutant adsorption and degradation in the downstream reach, resulting in improved water quality^43^. Water quality in the STR and DLR was generally fair, with particularly degraded conditions observed near the estuary (STR10), mainly due to elevated concentrations of COD_(Mn)_,TN, and TP exceeding water quality standards. In the LR Basin, frequent human activities led to substantial pollutant inputs, particularly from agricultural runoff and industrial discharges. These pollutants were difficult to fully purify and remove once they had entered the river, resulting in deteriorated water quality downstream^44^.

Based on the results of principal component analysis, it was evident that most of the water quality components in the DLR and STR exhibited significant seasonal variations. pH, TN, NO_3_^-^ and NH_4_^+^ values were significantly higher during the dry season compared to other periods. For instance, the mean TN concentrations during the dry season were 5.64 mg/L and 6.31 mg/L in the DLR and STR, markedly higher than those in the wet season (1.73 mg/L and 1.72 mg/L) and agricultural season (1.11 mg/L and 3.56 mg/L), respectively. During the dry season, the reduced river flow diminished the river’s dilution capacity. Residual alkaline fertilizers and nitrogen fertilizers, which were not absorbed by agricultural fields, entered the river through runoff, increasing the pH and nitrogen concentrations. Furthermore, although water volume decreased during the dry season, the continuous discharge of domestic and industrial wastewater from residents around the river also led to an elevation in nitrogen concentrations^45^. During the agricultural season, the concentrations of TP, PO_4_^3-^ and Chl-a in both DLR and STR increased significantly because of intensified agricultural fertilizer use^46^. The elevated phosphorus levels, particularly in the form of bioavailable phosphate, promoted phytoplankton growth, resulting in a marked increase in Chl-a concentration^47^. The primary components of water quality in the NLR during the wet season and fertilization period overlap significantly, indicating that agricultural runoff was a major source of these components. In the dry season, the primary components were more dispersed, suggesting that the sources of pollutants vary greatly between sampling sites. During the dry season, agricultural irrigation decreased, while industrial and domestic wastewater discharge became apparent under low flow conditions, resulting in increased dispersion of water quality across different sites^48^.

### Driving forces of river water quality

**Fig. 6:**
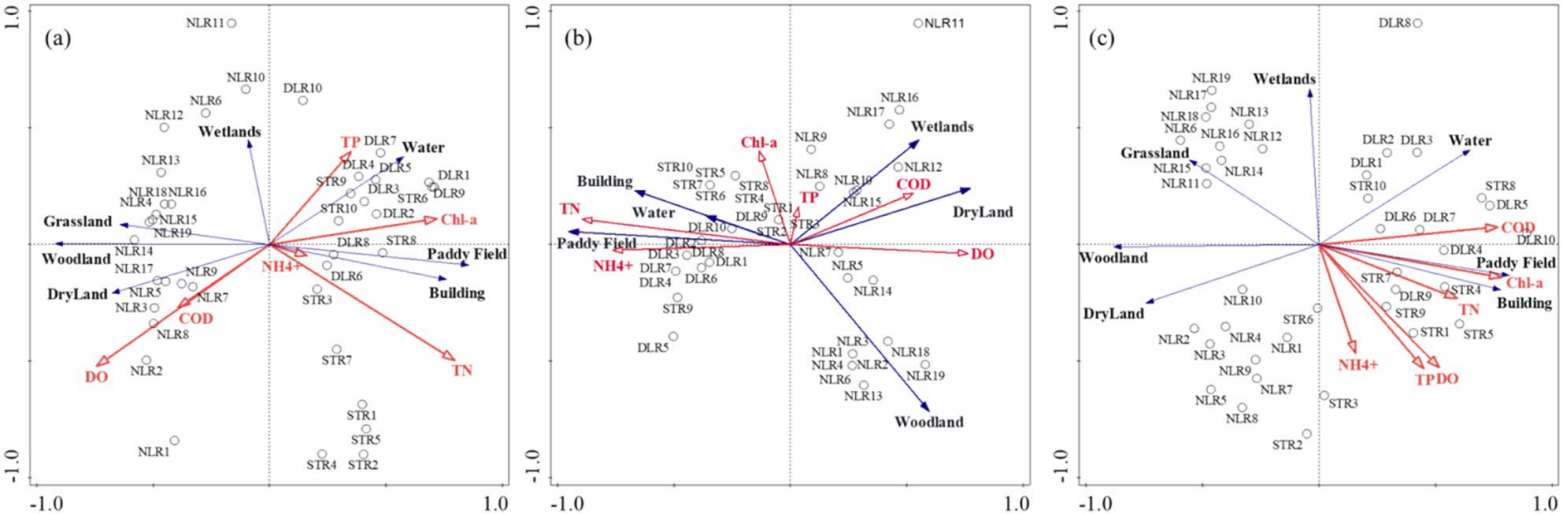
The RDA plots of water quality parameters and land use types during the (**a**) wet season, (**b**) dry season, and (**c**) agricultural season in DLR, STR and NLR.

Recognizing that spatial and temporal differences in water quality were often shaped by external environmental stressors, the potential influences of land use patterns and human activities were examined. Figure 6 presents the results of the redundancy analysis (RDA), illustrating the relationships between water quality parameters and land use types during the three observation periods. In both the dry and wet seasons (Fig. 6a, b), DO and COD were closely correlated with dry land and woodland, while nutrients and Chl-a were closely correlated with paddy fields and building areas in three studied rivers. During the agricultural season (Fig. 6c), all parameters showed stronger associations with crop land, especially paddy fields, reflecting the direct impact of fertilization activities on river water quality. Fertilization increased nutrient availability, promoting algal growth and leading to elevated organic matter accumulation in water bodies^49^. Studies have indicated that nutrient runoff and leaching from paddy fields were largely driven by irrigation practices and return flows, which compared to dryland farming, can exacerbate nutrient mobility and loss^50,51^. This further explained the dominance of paddy field agriculture in the DLR and STR basins, which led to overall higher nitrogen and phosphorus concentrations compared to the NLR (Section "Physico-biochemical parameters and nutrients"). Moreover, nutrients were also closely correlated with building areas in DLR and STR, while DO was closely correlated with woodland and wetland areas in NLR. The building areas in DLR and STR regions have formed impervious surfaces and the accumulation of nitrogen and phosphorus pollutants, which were intensively flushed into rivers during rainfall^52^. NLR had a larger proportion of woodland and wetland areas, accounting for 25.0% and 8.3%. These areas served as natural filters, which effectively reduced the sediment and pollutants in surface runoff and increased DO levels in rivers^53^.

Table S6 further displays the population density, industry, livestock density and GDP of counties involved in the drainage areas of DLR, STR, and NLR. In general, human-induced pollution activities were the least in NLR compared to DLR and STR, particularly characterized by relatively low population density and livestock density. The industrial production was primarily concentrated in Baoqing County in the upper and middle reaches of the basin (Fig S5), resulting in fair water quality status at NLR7—NLR10. Overall, water quality dynamics in the NLR were primarily driven by basin-wide agricultural land use and concentrated industrial activities in the upper to middle reaches. In contrast, DLR and STR basins had a high population and livestock density, which were consistent with the higher proportion of building areas. DLR and STR flow through the cities of Yingkou, Anshan and Panjin (Fig S5), which have been greatly impacted by dense population and intensive industrial activities. In particular, the livestock breeding was intense in STR with livestock density in Tai’an reaching 1868 heads/km^2^. Wastewater from intensive livestock and poultry farming contributed to the high levels of nitrogen and phosphorus^54^. Additionally, industries in STR included oil extraction, chemical plants, and dye factories, which produced a large amount of organic pollutants^25^. These factors ultimately resulted in the unsatisfactory water quality status in STR, particularly at STR10 near the estuary (refer to Section "CCME-WQI results").

Land use and human activities exerted significant influences on river water quality through multiple pathways. Agricultural land, especially when dominated by intensive cropping or paddy fields, often led to increased nutrient loads due to fertilizer application, irrigation, and surface runoff. Urban and industrial areas contributed a wide range of pollutants, primarily through wastewater discharge and stormwater runoff. High livestock density acted as key source of nitrogen and phosphorus pollution. Changes in land use altered the hydrological response of catchments, affecting the transport and accumulation of pollutants. Therefore, effective water quality conservation requires an integrated approach addressing land use configuration, agricultural intensity, and both point and non-point source pollution.

### Source analysis of heavy metals

The presence of heavy metals in surface water is closely related to human health. Our investigation showed that the heavy metal content in NLR may pose health risks to residents, primarily due to the carcinogenic substance As and non-carcinogenic substances like Pb, Mn, Mo and Se (section "Human health risks posed by heavy metals"). Crop planting was identified as the primary activity affecting the water quality in NLR. Fertilizers and pesticides used in agriculture often contained heavy metals^55^, which were likely to enter the river through irrigation return flow and agricultural surface runoff, becoming a major source of heavy metals in the river. Song et al.^56^ found that the concentrations of Pb and Ni in agricultural land around NLR exceeded background values. In addition, coal mines and dye factories were distributed in the upper and middle reaches of NLR, producing heavy metals like As, Zn, Cu and Ni and impacting nearby water bodies^13^. Notably, the elevated arsenic risk in the NLR during wet and agricultural seasons was likely attributed to the combined effects of atmospheric deposition and seasonal hydrological influences. Mining and coal combustion activities released gaseous As compounds that entered surface waters through wet and dry atmospheric deposition^57^. During the wet season, increased precipitation and surface runoff intensified the leaching and mobilization of As from mining waste, contaminated soils, and river sediments^58^. In the agricultural season, irrigation practices further facilitated As mobilization. Intensive groundwater extraction for irrigation induced the upward migration of As-contaminated groundwater into surface water systems, particularly in areas where shallow aquifers had been historically affected by mining activities^59^. The STR estuary hosted the nation’s third-largest oilfield, where heavy metals such as Ni and Zn were transported upstream via tidal intrusion^60^. In addition, the presence of As and Ni in STR may have originated from sediment release^61^. Children were more vulnerable to heavy metal risks compared to adults and required more protection.

## Conclusions

This study conducted a comparative analysis of water quality characteristics and human health risks posed by heavy metals in three rivers of the Songliao River Basin: the Daliao River, the Shuangtaizi River, and the Naoli River, under varying seasons and human-induced land use patterns. The comprehensive analysis of 22 water quality parameters revealed that DLR and STR were more affected by nitrogenous nutrients and heavy metals (Zn, Mn, Pb), while NLR was primarily affected by TP. The distinct land use patterns played a crucial role in determining water quality. DLR and STR basins were primarily dominated by paddy field and building areas, while NLR basin was mainly characterized by dryland, wetlands, and woodland. The downstream regions of DLR and STR experienced increased pollution due to domestic sewage and industrial activities, while NLR benefited from natural purification processes provided by riparian vegetation and wetlands. Water quality components in both DLR and STR showed considerable variations across different periods, driven by industry, agriculture, and urbanization. In contrast, water quality in NLR during the wet season and agricultural season was heavily influenced by agricultural activities, highlighting the critical role of farming practices in this region. The study revealed that heavy metal risks, particularly from carcinogenic As, posed substantial health concerns in STR and NLR. Children consistently faced higher risks than adults due to their lower immune resistance. Major sources of these heavy metals included fertilizers and pesticides used in agriculture, as well as industrial activities such as mining and oil extraction. Non-carcinogenic metals, on the other hand, exhibited relatively low risk values and did not pose immediate health threats. Therefore, controlling As levels in STR and NLR was crucial for safeguarding public health. In summary, targeted land use management and environmental policies are essential to balancing industrial and agricultural developments with ecological protection. Effective strategies must integrate land use considerations to safeguard water resources and mitigate health risks associated with pollution in these river basins.

## Supplementary Information

Supplementary Information.

## Data Availability

All data generated or analysed during this study are included in this published article [and its supplementary information files].
