# Supplementary material for: Spatial–temporal variations of river water quality under human-induced land use changes in large river basins
Source: Sci Rep. 2025 Oct 22;15:36955. doi: 10.1038/s41598-025-20876-z (PMC12546616; doi:10.1038/s41598-025-20876-z)
Supplement: Supplementary file 1 — Supplementary Information. [file 41598_2025_20876_MOESM1_ESM.docx]

**Supplementary Material (SM)**

**Spatial-temporal variations of river water quality under human-induced land use changes in large river basins**

Xiaojing Zhang^1^, Bing Yu^1,*^, Zhuohang Xin^1^, Ming Cong^1^, Chi Zhang^1^

^1^*School of Infrastructure Engineering, Dalian University of Technology, Dalian 116024, China*

*Corresponding authors: yubing@dlut.edu.cn (B.Yu).


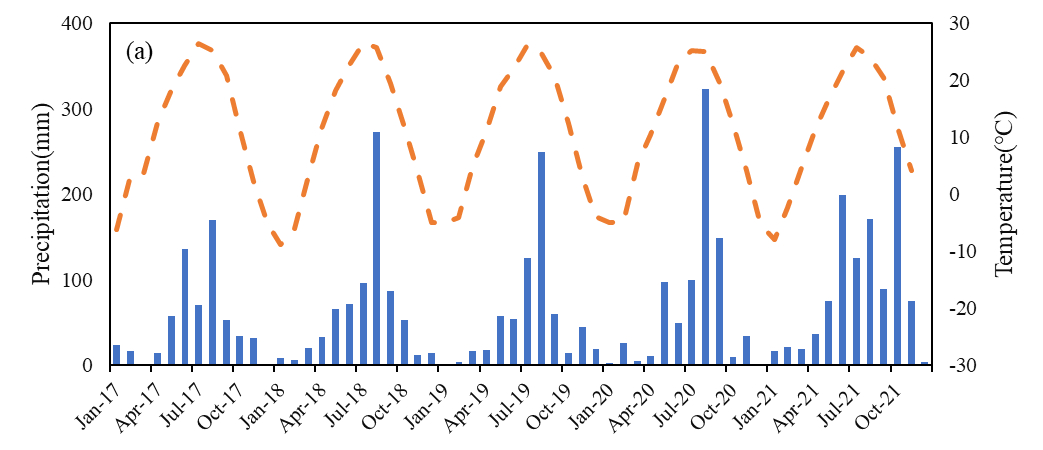


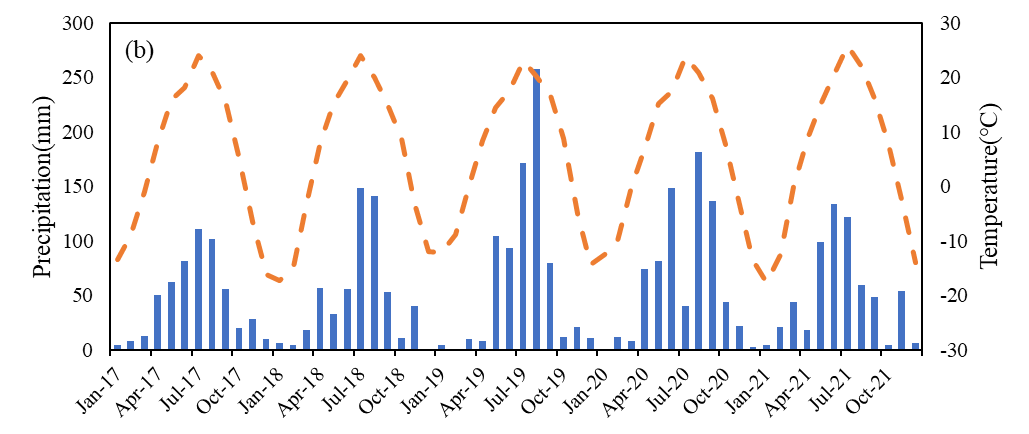


Fig. S1. Distribution of monthly precipitation and temperature from 2017 to 2021 in (a) the Liao River basin and (b) the Naoli River basin.


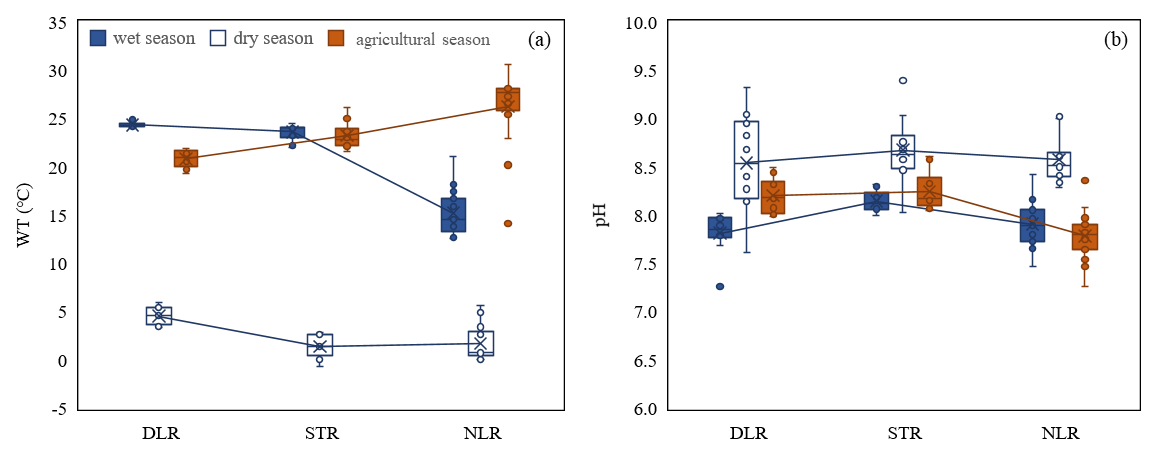


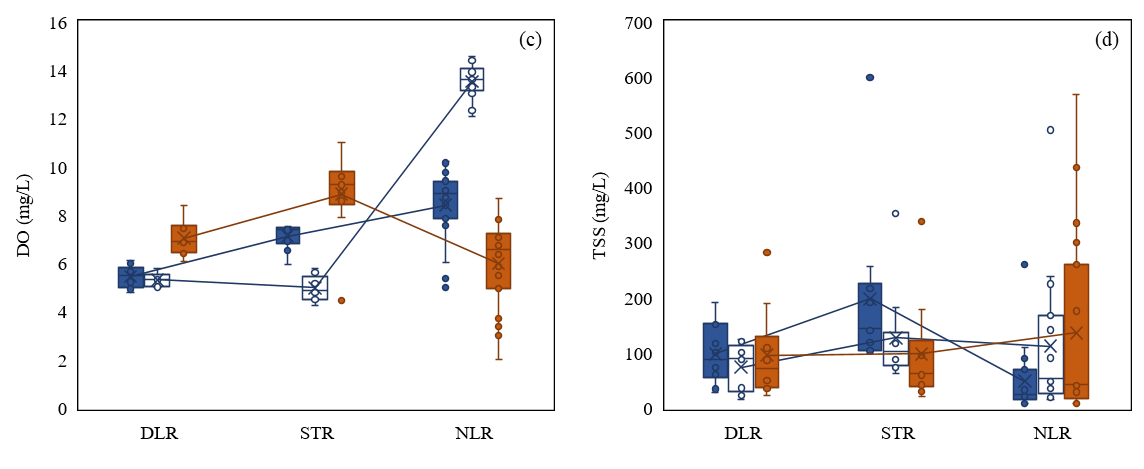


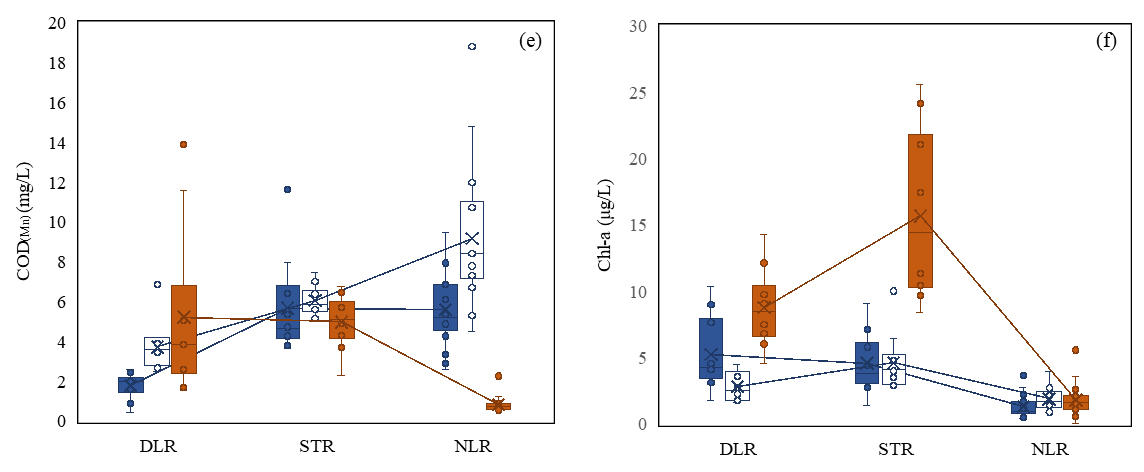


Fig. S2. Box charts of temporal variations of (a) WT, (b) pH, (c) DO, (d) TSS (e) COD_(Mn)_ and (f) Chl.a in the DLR, STR and NLR.

In Fig.S2a, it can be seen that water temperature (WT) of these rivers was close in wet season and agricultural season, ranged between 15 and 25℃, while it was lower in dry season, ranged between 0 and 5℃. pH in the DLR, STR and NLR showed weakly alkaline, and it was affected by WT. As WT increased, pH decreased. pH was higher in dry season (8.5 ± 0.9, 8.7 ± 1.4 and 8.6 ± 0.7 in the DLR, STR and NLR, respectively) and lower in wet season (7.8 ± 0.6, 8.1 ± 0.2 and 7.9 ± 0.5 in the DLR, STR and NLR, respectively). TSS fluctuated without specific seasonal variability in the three rivers, with the mean value of 89.51 mg/L, 141.34 mg/L and 99.04 mg/L in the DLR, STR and NLR,respectively.
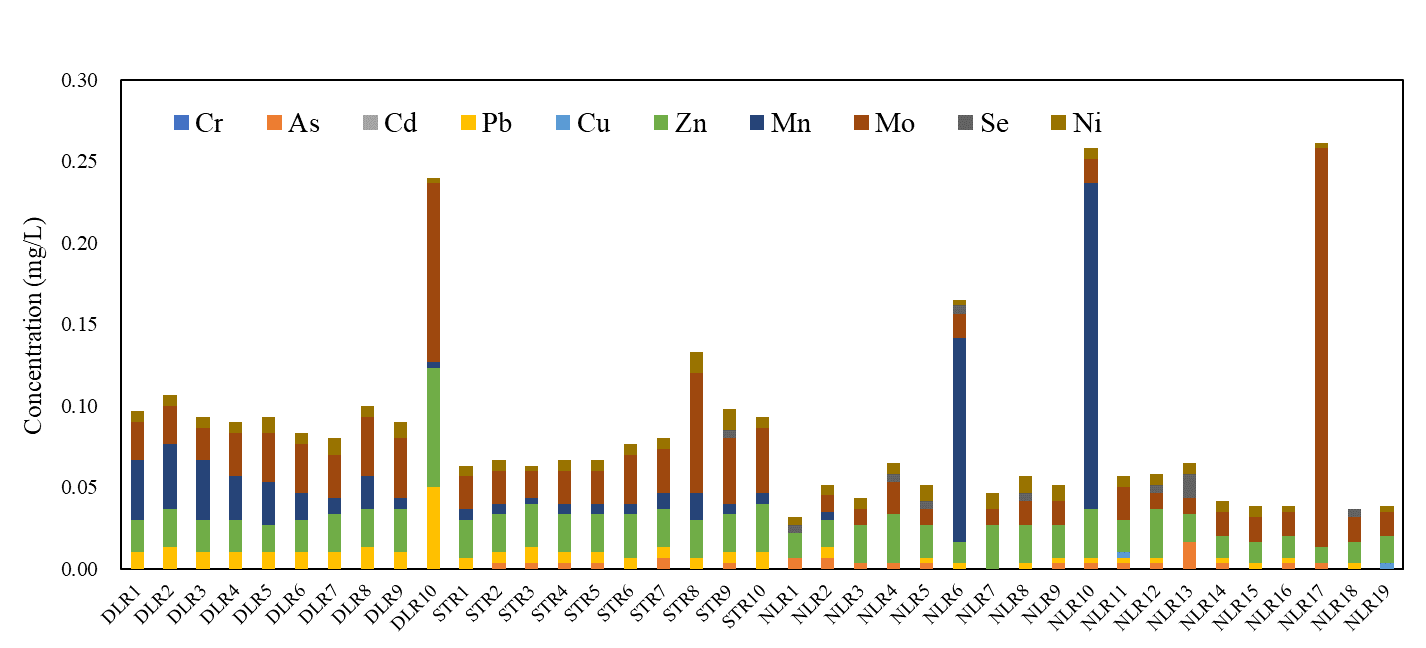


Fig. S3 Stacked bar chart of annual average heavy metal concentrations at each point.

Table S1 Correlation Analysis of Comprehensive Water Quality Indicators.

| Pearson Correlation | | | | | | | | | | | | | | | | | | | | |
| --- | --- | --- | --- | --- | --- | --- | --- | --- | --- | --- | --- | --- | --- | --- | --- | --- | --- | --- | --- | --- |
|  | WT | pH | DO | TSS | COD | Chl-a | TN | NH_4_^+^ | NO_2_^-^ | NO_3_^-^ | TP | PO_4_^3-^ | As | Pb | Cu | Zn | Mn | Mo | Se | Ni |
| WT | 1 |  |  |  |  |  |  |  |  |  |  |  |  |  |  |  |  |  |  |  |
| pH | -.650** | 1 |  |  |  |  |  |  |  |  |  |  |  |  |  |  |  |  |  |  |
| DO | -.370** | .337** | 1 |  |  |  |  |  |  |  |  |  |  |  |  |  |  |  |  |  |
| TSS | 0.121 | 0.078 | -0.03 | 1 |  |  |  |  |  |  |  |  |  |  |  |  |  |  |  |  |
| COD | -.533** | .447** | .556** | .276** | 1 |  |  |  |  |  |  |  |  |  |  |  |  |  |  |  |
| Chl-a | -.295** | .349** | -.235* | 0.057 | 0.13 | 1 |  |  |  |  |  |  |  |  |  |  |  |  |  |  |
| TN | -.437** | .491** | -.361** | 0.09 | 0.022 | .429** | 1 |  |  |  |  |  |  |  |  |  |  |  |  |  |
| NH_4_^+^ | -.322** | .342** | -.277** | -0.08 | -0.1 | 0.163 | .709** | 1 |  |  |  |  |  |  |  |  |  |  |  |  |
| NO_2_^-^ | 0.041 | 0.135 | -0.16 | -0.01 | -0.08 | 0.151 | .367** | 0.17 | 1 |  |  |  |  |  |  |  |  |  |  |  |
| NO_3_^-^ | -.358** | .433** | -.410** | 0.17 | 0.016 | .390** | .942** | .606** | .388** | 1 |  |  |  |  |  |  |  |  |  |  |
| TP | .202* | 0.043 | 0.021 | .213* | 0.02 | 0.092 | 0.163 | 0.098 | 0.117 | 0.075 | 1 |  |  |  |  |  |  |  |  |  |
| PO_4_^3-^ | .188* | -0.01 | -0.13 | 0.081 | -0.09 | -0.13 | 0.16 | 0.127 | .235* | 0.12 | .600** | 1 |  |  |  |  |  |  |  |  |
| As | .332** | -0.07 | -0.05 | .273** | -.291** | -0.01 | -0.1 | 0.012 | -0.04 | -0.12 | 0.119 | 0.029 | 1 |  |  |  |  |  |  |  |
| Pb | -.492** | .355** | -0.17 | -0.04 | 0.105 | .216* | .497** | .569** | 0.111 | .568** | -0.07 | 0.097 | -0.11 | 1 |  |  |  |  |  |  |
| Cu | -0.03 | -0.17 | -0.04 | -0.11 | 0.035 | -0.07 | -0.12 | -0.08 | -0.09 | -0.13 | -0.1 | -0.06 | -0.05 | -0.05 | 1 |  |  |  |  |  |
| Zn | -.449** | .316** | -.249** | 0.092 | -0.05 | .338** | .556** | .546** | 0.026 | .550** | 0.063 | 0.118 | 0.111 | .840** | -0.1 | 1 |  |  |  |  |
| Mn | -.271** | 0.053 | -0.14 | -0.17 | -0.17 | -0.05 | .276** | .377** | -0.03 | .216* | -0.14 | -0.07 | -0.06 | .221* | -0.05 | .244* | 1 |  |  |  |
| Mo | -0.01 | 0.024 | -0.09 | 0.016 | 0.067 | 0.02 | 0.103 | 0.066 | 0.083 | 0.123 | 0.033 | 0.073 | -0.05 | 0.105 | -0.06 | 0.082 | 0.001 | 1 |  |  |
| Se | .289* | -0.21 | -0.14 | .357** | -.278* | -0.06 | -0.18 | -0.15 | -0.17 | -0.14 | -0.03 | -0.06 | .671** | -0.14 | -0 | 0.093 | 0.002 | -0 | 1 |  |
| Ni | -.319** | .205* | 0.057 | 0.097 | .364** | -0.05 | 0.142 | 0.005 | 0.039 | .233* | -.284** | -0.05 | -.313** | 0.157 | 0.065 | -0.03 | 0.1 | 0.136 | -.230* | 1 |

** Correlation is significant at the 0.01 level (2-tailed).

* Correlation is significant at the 0.05 level (2-tailed).

Table S1 excludes Cr and Cd, because the concentrations of Cr and Cd at all sampling points were below the detection limit of 0.01 mg/L.

Table S2 The Kaiser-Meyer-Olkin (KMO) test and Bartlett's test.

| KMO and Bartlett's Test of the DLR | |
| --- | --- |
| Kaiser-Meyer-Olkin Measure of Sampling Adequacy. | 0.629 |
| Bartlett's Test of Sphericity (Sig.) | 0.000 |

| KMO and Bartlett's Test of the STR | |
| --- | --- |
| Kaiser-Meyer-Olkin Measure of Sampling Adequacy. | 0.623 |
| Bartlett's Test of Sphericity (Sig.) | 0.000 |

| KMO and Bartlett's Test of the NLR | |
| --- | --- |
| Kaiser-Meyer-Olkin Measure of Sampling Adequacy. | 0.671 |
| Bartlett's Test of Sphericity (Sig.) | 0.000 |


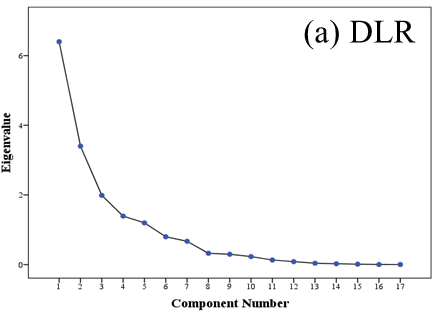

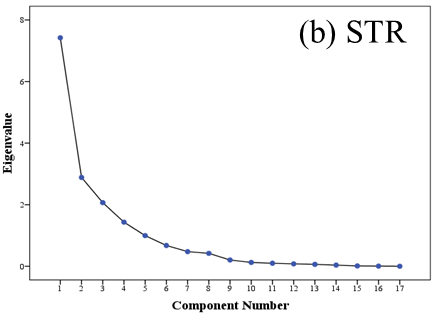

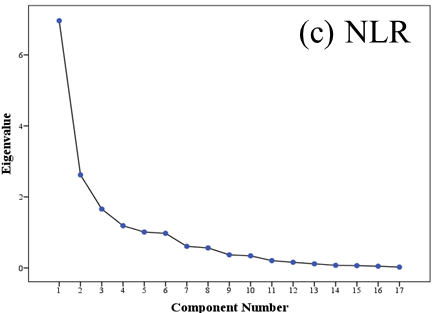


Figure S4 The scree plots of PCA in the DLR, STR, and NLR.

Table S3 The calculated F, CCME-WQI value and river categorization in each sampling site.

| Sampling sites | F1 | F2 | F3 | CCME WQI | Category |
| --- | --- | --- | --- | --- | --- |
| DLR1 | 10.00 | 20.00 | 26.16 | 80.13 | good |
| DLR2 | 10.00 | 16.67 | 28.27 | 80.19 | good |
| DLR3 | 10.00 | 20.00 | 24.82 | 80.71 | good |
| DLR4 | 20.00 | 16.67 | 38.63 | 73.10 | fair |
| DLR5 | 20.00 | 23.33 | 39.28 | 71.21 | fair |
| DLR6 | 20.00 | 20.00 | 33.63 | 74.63 | fair |
| DLR7 | 20.00 | 16.67 | 33.27 | 75.61 | fair |
| DLR8 | 20.00 | 13.33 | 29.17 | 78.18 | fair |
| DLR9 | 20.00 | 16.67 | 35.37 | 74.64 | fair |
| DLR10 | 30.00 | 23.33 | 31.97 | 71.33 | fair |
| STR1 | 30.00 | 20.00 | 35.08 | 70.96 | fair |
| STR2 | 30.00 | 23.33 | 35.06 | 70.15 | fair |
| STR3 | 30.00 | 23.33 | 31.06 | 71.66 | fair |
| STR4 | 30.00 | 26.67 | 37.96 | 68.10 | fair |
| STR5 | 40.00 | 26.67 | 38.49 | 64.44 | fair |
| STR6 | 30.00 | 20.00 | 36.07 | 70.55 | fair |
| STR7 | 40.00 | 26.67 | 38.08 | 64.59 | fair |
| STR8 | 30.00 | 30.00 | 34.55 | 68.41 | fair |
| STR9 | 30.00 | 20.00 | 37.08 | 70.14 | fair |
| STR10 | 40.00 | 33.33 | 43.72 | 60.74 | marginal |
| NLR1 | 10.00 | 10.53 | 15.94 | 87.55 | good |
| NLR2 | 10.00 | 10.34 | 4.43 | 91.31 | good |
| NLR3 | 20.00 | 10.34 | 16.04 | 84.04 | good |
| NLR4 | 20.00 | 13.79 | 18.71 | 82.29 | good |
| NLR5 | 30.00 | 24.14 | 31.75 | 71.18 | fair |
| NLR6 | 0.00 | 17.24 | 11.57 | 88.01 | good |
| NLR7 | 30.00 | 24.14 | 29.43 | 72.02 | fair |
| NLR8 | 20.00 | 21.43 | 27.08 | 76.96 | fair |
| NLR9 | 30.00 | 24.14 | 29.72 | 71.92 | fair |
| NLR10 | 30.00 | 17.24 | 21.41 | 76.51 | fair |
| NLR11 | 10.00 | 10.34 | 4.78 | 91.25 | good |
| NLR12 | 20.00 | 17.24 | 11.30 | 83.42 | good |
| NLR13 | 10.00 | 6.90 | 3.79 | 92.65 | good |
| NLR14 | 0.00 | 3.45 | 0.74 | 97.96 | excellent |
| NLR15 | 0.00 | 3.45 | 1.35 | 97.86 | excellent |
| NLR16 | 0.00 | 6.90 | 2.08 | 95.84 | excellent |
| NLR17 | 0.00 | 10.34 | 2.31 | 93.88 | good |
| NLR18 | 0.00 | 13.79 | 3.68 | 91.76 | good |
| NLR19 | 0.00 | 10.34 | 3.25 | 93.74 | good |

Table S4 Toxicological parameters of the heavy metals/mg·(kg·d)^-1^.

| Element | Carcinogen(Qig) | non-carcinogen(RfDjg) |
| --- | --- | --- |
| Cr | 41 |  |
| As | 15 |  |
| Cd | 6.1 |  |
| Pb |  | 0.0014 |
| Cu |  | 0.005 |
| Zn |  | 0.3 |
| Mn |  | 0.14 |
| Mo |  | 0.005 |
| Se |  | 0.005 |
| Ni |  | 0.02 |

United States Environmental Protection Agency（USEPA）

Table S5 Human health risk values of each heavy metal in different periods/year^-1^.

| Period | Human | Rivers | Cr | As | Cd | Pb | Cu | Zn | Mn | Mo | Se | Ni |
| --- | --- | --- | --- | --- | --- | --- | --- | --- | --- | --- | --- | --- |
| Wet season | Adult | DLR | 0 | 0 | 0 | 0 | 0 | 0 | 0 | 2.06E-09 | 0 | 2.32E-10 |
|  |  | STR | 0 | 0 | 0 | 0 | 0 | 0 | 1.10E-11 | 3.70E-09 | 0 | 3.34E-10 |
|  |  | NLR | 0 | 4.07E-06 | 0 | 0 | 1.06E-10 | 0 | 7.97E-11 | 3.54E-09 | 0 | 2.45E-10 |
|  | Child | DLR | 0 | 0 | 0 | 0 | 0 | 0 | 0 | 2.38E-09 | 0 | 2.68E-10 |
|  |  | STR | 0 | 0 | 0 | 0 | 0 | 0 | 1.28E-11 | 4.28E-09 | 0 | 3.87E-10 |
|  |  | NLR | 0 | 4.71E-06 | 0 | 0 | 1.22E-10 | 0 | 9.22E-11 | 4.09E-09 | 0 | 2.83E-10 |
| Agricultural season | Adult | DLR | 0 | 0 | 0 | 0 | 0 | 3.43E-12 | 0 | 4.42E-09 | 0 | 7.72E-11 |
|  |  | STR | 0 | 3.85E-05 | 0 | 0 | 0 | 5.14E-12 | 7.35E-12 | 2.37E-09 | 0 | 5.14E-11 |
|  |  | NLR | 0 | 7.30E-05 | 0 | 0 | 0 | 6.26E-11 | 0 | 0 | 5.44E-10 | 0 |
|  | Child | DLR | 0 | 0 | 0 | 0 | 0 | 3.97E-12 | 0 | 5.12E-09 | 0 | 8.93E-11 |
|  |  | STR | 0 | 4.45E-05 | 0 | 0 | 0 | 5.95E-12 | 8.50E-12 | 2.74E-09 | 0 | 5.95E-11 |
|  |  | NLR | 0 | 8.44E-05 | 0 | 0 | 0 | 7.25E-11 | 0 | 0 | 6.30E-10 | 0 |
| Dry season | Adult | DLR | 0 | 0 | 0 | 1.63E-08 | 0 | 1.34E-10 | 2.47E-10 | 4.76E-09 | 0 | 2.59E-10 |
|  |  | STR | 0 | 1.55E-05 | 0 | 8.13E-09 | 0 | 1.22E-10 | 6.65E-11 | 3.41E-09 | 1.03E-10 | 2.07E-10 |
|  |  | NLR | 0 | 0 | 0 | 2.67E-09 | 0 | 3.73E-11 | 5.13E-11 | 1.78E-09 | 0 | 2.44E-10 |
|  | Child | DLR | 0 | 0 | 0 | 1.88E-08 | 0 | 1.56E-10 | 2.86E-10 | 5.50E-09 | 0 | 2.99E-10 |
|  |  | STR | 0 | 1.79E-05 | 0 | 9.40E-09 | 0 | 1.42E-10 | 7.69E-11 | 3.95E-09 | 1.20E-10 | 2.39E-10 |
|  |  | NLR | 0 | 0 | 0 | 3.09E-09 | 0 | 4.32E-11 | 5.93E-11 | 2.06E-09 | 0 | 2.83E-10 |

Table S6 Population and socioeconomic information of DLR, STR and NLR.

| River | City | County | Population Density (people/km²) | Number of Industries | Livestock Density (heads/km²) | GDP (CNY/person) |
| --- | --- | --- | --- | --- | --- | --- |
| DLR | Yingkou | Dashiqiao | 390 | 11 | 106 | 50139 |
|  |  | Xishi | 8000 | 39 | 107 | 47037 |
|  |  | Zhanqian | 852 | 34 | 107 | 42025 |
| STR | Panjin | Dawa | 215 | 70 | 298 | 31700 |
|  |  | Panshan | 136 | 72 | 295 | 73400 |
|  |  | Xinglongtai | 1645 | 69 | 52 | 31675 |
|  | Anshan | Tai'an | 262 | 66 | 1868 | 88096 |
|  |  | Haicheng | 494 | 2 | 154 | 67599 |
| NLR | Shuangyashan | Baoqing | 29 | 41 | 43 | 52258 |
|  |  | Raohe | 22 | 0 | 17 | 35133 |
|  | Qitaihe | Qiezihe | 26 | 1 | 19 | 25974 |


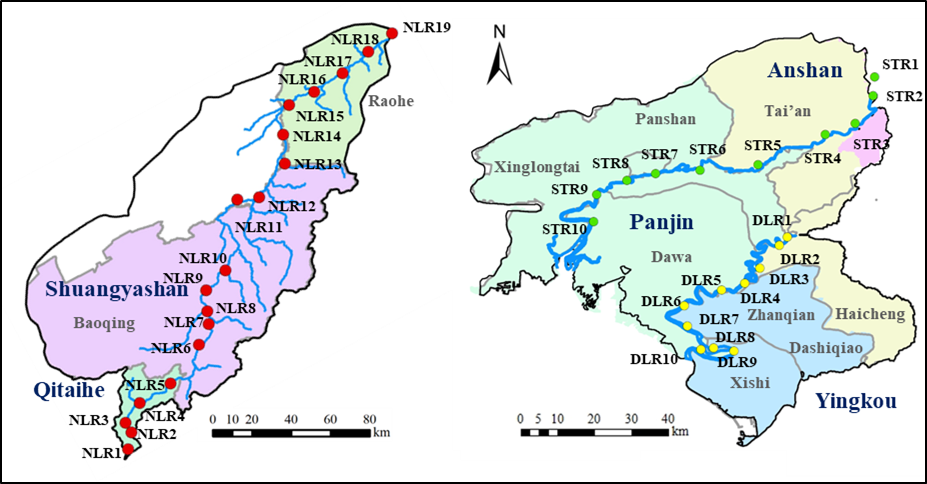


Figure S5 Map of administrative cities and counties in STR, DLR and NLR.
